# Changes in self-efficacy in Japanese school-age children with and without high autistic traits after the Universal Unified Prevention Program: a single-group pilot study

**DOI:** 10.1186/s13034-021-00398-y

**Published:** 2021-08-26

**Authors:** Takuya Oka, Shin-ichi Ishikawa, Aya Saito, Kazushi Maruo, Andrew Stickley, Norio Watanabe, Hiroki Sasamori, Toshiki Shioiri, Yoko Kamio

**Affiliations:** 1 Department of Psychiatry and Psychotherapy, Gifu University Graduate School of Medicine, Gifu, Japan; 2 Department of Preventive Intervention for Psychiatric Disorders, National Institute of Mental Health, National Center of Neurology and Psychiatry, Tokyo, Japan; 3 Faculty of Psychology, Doshisha University, Kyoto, Japan; 4 Department of Biostatistics, Faculty of Medicine, University of Tsukuba, Ibaraki, Japan; 5 Faculty of Core Research, Ochanomizu University, Tokyo, Japan; 6 Department of Psychiatry, Soseikai General Hospital, Kyoto, Japan; 7 Center for Promoting Education for Persons with Developmental Disabilities, National Institute of Special Needs Education, Kanagawa, Japan; 8 Institute for Education and Human Development, Ochanomizu University, Tokyo, Japan; 9 Social Medical Corporation Seisenkai, Holy cross hospital, Gifu, Japan

**Keywords:** Self-efficacy, Mental health, Children, Autistic traits, Cognitive- behavioral therapy, School-based program, Mainstream classes

## Abstract

**Background:**

Research has shown the efficacy of school-based programs for mental health problems in children. However, few studies have focused on the strengths of children, such as resilience, which is essential in preventing mental health problems. Moreover, no research has investigated the effect of a universal school-based program on children with increased autistic traits in mainstream classes. We examined the changes in children's self-efficacy, social skills, and general mental health after the implementation of a newly developed universal program, the Universal Unified Prevention Program for Diverse Disorders (Up2-D2), and whether similar changes occurred in children with and without higher autistic traits.

**Methods:**

To assess possible changes associated with the program, questionnaires were collected from 396 children (207 boys and 189 girls) aged 9–12 years old before (T1), immediately after (T2), and three months after (T3) the implementation of the program.

**Results:**

Results from a linear mixed-effects model showed a significant increase in children's self-efficacy at T2 (adjusted difference 0.49, 95% CI 0.03–0.94; p < 0.05) and T3 (0.78, 95% CI 0.32–1.23; p < 0.001). There were also significant positive changes in social skills and general mental health. Similar changes were observed in children with high autistic traits. Autistic traits at T1 did not contribute to the degree of change in self-efficacy.

**Conclusions:**

Our pilot study suggests that a universal program has the potential to promote positive attitudes and mental health in both at-risk and not-at-risk children.

**Supplementary Information:**

The online version contains supplementary material available at 10.1186/s13034-021-00398-y.

## Introduction

Tackling children's mental health problems is an urgent global issue. It is estimated that 10–20% of children have one or more mental health problems [1], and these mental health problems are also known to affect a wide range of outcomes in adulthood, even if the problems are subthreshold [2]. Given the evidence for the efficacy of preventive interventions for mental health problems in children [3], schools are expected to play a role in recognizing children’s mental health needs and serving as a place to implement prevention programs [4].

School-based universal programs include all children enrolled in school, whereas selective and indicated approaches target only high-risk children [5]. Regarding the efficacy of school-based depression prevention programs using universal or selective approaches, a previous meta-analysis evaluated 32 programs and found that only the selective approach resulted in a prevention effect [6]. In addition, a network meta-analysis evaluating 137 randomized controlled trials (RCT) to reduce anxiety and depression in 4–18 year olds concluded that there was insufficient evidence of their effectiveness [7]. These studies, however, may have drawn somewhat premature conclusions on the efficacy of interventions since the benefits of school environments in promoting resilience have still not been fully evaluated [8]. In particular, there is a need to evaluate the potential effects of school-based prevention programs in a more comprehensive manner, not just in terms of symptom improvement.

Dray and colleagues [9] focused on the efficacy of universal school-based programs that aimed to heighten resilience in individuals aged 5–18 years old. Their meta-analysis evaluated effect sizes for seven outcomes (anxiety, depression, hyperactivity, behavioral problems, internalizing problems, externalizing problems, and general psychological stress) with data from 57 RCTs, with small effects being found for 4 of the 7 outcomes (depression, internalizing problems, externalizing problems, and general psychological stress) (Standardized Mean Difference; SMD = − 0.08, − 0.21, − 0.18, − 0.11) [9]. Fenwick-Smith and colleagues [10] selected studies, including non-randomized trials, that measured resilience or related factors such as self-efficacy or coping skills as outcomes and conducted a systematic review of studies of universal programs for elementary school children (aged 5–12 years old). Of the 11 studies examined, 10 produced positive results in terms of student resilience and related factors [10]. Although prior research has thus suggested that universal programs may have a beneficial effect, as yet, very few studies have assessed resilience or self-efficacy as a primary outcome.

Ishikawa and colleagues [11] have developed a new school-based universal prevention program—*the Universal Unified Prevention Program for Diverse Disorders* (Up2-D2), which targets transdiagnostic mental health problems in primary and secondary school students aged 8–15 years old. Specifically, the Up2-D2 is characterized by five main features: (1) a transdiagnostic approach, (2) a positive orientation, (3) a cartoon story, (4) a teaching plan for teachers, and (5) interpersonal practice (inter-peer interactive activities), and is designed to integrate common components of cognitive behavior therapy (CBT) such as psychoeducation, behavioral activation, social skills training, relaxation, cognitive restructuring, graded exposure, and problem-solving. These components are modified and tailored to fit a school's curriculum and educational format, allowing classroom teachers to implement the program in the classroom. In relation to this, a feasibility study showed that there was a tendency for children’s self-efficacy to gradually increase through the program. In addition, the overall fidelity of the program implemented by teachers was judged as being sufficient (76.2%) with children exhibiting stable enjoyment, comprehension, attainment, and application through the sessions. These findings support the idea that the program is feasible [11].

Considering individual differences, the effect of interventions on high-risk children who have developmental disorder symptoms/traits and are enrolled in mainstream classes is an important, but overlooked topic. It has been reported that the estimated prevalence of autism spectrum disorder (ASD) is 1.89% in mainstream school classes [12]. Furthermore, previous studies have found that additional mental health problems commonly occur in children with ASD [13, 14], and that those with a higher level of autistic traits have a greater risk of additional mental health problems [15, 16]. Importantly, there is some evidence that autistic traits may have long-term negative effects. For example, a recent longitudinal community-based study showed that emotional symptoms and peer problems in schoolchildren at age 7 were predicted by higher autistic traits assessed at age 5 [17]. Another study found that higher depressive symptoms in children with ASD and autistic traits who were 10 years old were still present when they were 18 years old [18]. Preventive interventions may be efficacious for children both with and without a higher level of autistic traits. However, to the best of our knowledge, as yet, no research has examined whether universal programs have a similar impact on all children in mainstream classes, including those with higher autistic traits.

Given this, we designed a pilot study where the main objectives were to:

1. Evaluate behavioral changes that occur in children in terms of self-efficacy, social skills and mental health outcomes when they receive the Up2-D2 program in mainstream classes.

2. Assess the impact of autistic traits on all outcomes after the program.

3. Determine whether individual factors such as age, autistic traits, and baseline mental health influence behavioral changes in these children.

## Methods

### Study design and setting

This study employed a single-group pre- and post-test design. We evaluated the effect of the Up2-D2 program on the self-efficacy, social skills, and general mental health of children in Japanese elementary schools.

### Recruitment and participants

The authors approached the local government boards of education in three prefectures (Kyoto, Gifu, Saitama) in Japan to explain the study plan. All agreed to conduct the program as part of their regular curricula for 4th to 6th grade children, and 8 elementary schools were nominated by them to participate in the study. The school principals agreed to their schools participating after they were provided with the details of the program. The principal of each school then sent a letter to all the parents of children in the 4th to 6th grades informing them about the study. The parents of 396 children (a 55% response rate) in the 8 schools gave written consent for their children to participate in the study.

### Procedure

The intervention program was implemented by each classroom teacher once a week as part of the regular curriculum from September 2016 to March 2017. Before the program began, the teachers received one day of training provided by one of the authors. The teaching plans and visual materials to be used in the classroom were provided to them before the training started. After the training was completed, teachers were instructed to practice by themselves using a training DVD that was given to them.

Questionnaire assessments were conducted at three time points: before the start of the intervention (T1), immediately after the end of the program (T2), and 3 months after the end of the intervention (T3). Children completed questionnaires during classes at T1, T2, and T3. To ensure that children could understand each question and answer it properly, the teachers explained all the question sentences in the class and allowed children sufficient time to answer them. Furthermore, teachers stated orally that the children’s answers would remain confidential and would not be shared with either parents or other teachers. Each classroom teacher rated 10 children (5 boys, 5 girls) who were randomly chosen from among those children whose parents had provided written consent according to a predetermined procedure. When the parents of fewer than 10 children provided consent, teachers rated all of the children for whom parents had provided consent. The completed questionnaires were sent from the schools to the research team. After completing questionnaires at home about their children, the parents also sent them to the research team (Additional file 1).

The study procedures were in accordance with conventional ethical standards and approved by the Ethics Committee of the National Center of Neurology and Psychiatry, Tokyo, Japan (A2016-035). Only data that were obtained through the consent process were analyzed in the current study.

### Intervention

The Up2-D2 was used in the current study. It consists of 12 sessions, each lasting about 45 min. The details of the program components are shown in Additional file 2.

### Intervention fidelity

Eleven sessions excluding the final session (review and conclusion) were recorded using an integrated chip (IC) recorder. We randomly extracted 9 sessions per school, resulting in 72 sessions (27.3%), for which we assessed fidelity. Trained research assistants rated recorded sessions using a checklist relating to the main topics to be covered in each session. The overall fidelity was on average 79.8% (SD = 5.63, Range 70.2–80.7).

## Measures

### Primary outcome measure

#### Self-efficacy

We used the self-rated General Self-Efficacy Scale for Children-Revised (GSESC-R; [19]), that has been standardized for Japanese children. Factor analysis extracted two factors, ‘sensitivity to failure experiences’ (9 items) and a ‘positive attitude’ (9 items) [19]. The latter ‘positive attitude’ factor (e.g. ‘When I make plans, I am certain I can make them work’, ‘When I have something complicated to do, I will manage to achieve it’, ‘I am a self-reliant person’) was used in this study. Each item is scored on a 4-point scale (range 9–36). Higher scores indicate greater self-efficacy. The internal consistency of the ‘positive attitude’ factor has been shown to be high (Cronbach’s α = 0.81) among Japanese primary school children [19]. The Cronbach's α in our study was 0.77.

### Secondary outcome measures

#### Social skills and problem behaviors

The Children’s Social Skills Scale (CSSS; [20]) was used in the current study. The CSSS is a teacher-rated 37-item scale consisting of 25 items that assess social skills (social initiation, academic performance, self-control, peer reinforcement, and compliance) and 12 items that assess problem behaviors (externalizing behavior and internalizing behavior). Each item is rated on a five-point scale with the total scale score ranging from 37 to 185. Higher scores indicate that children have better social skills and engage in more favorable behavior. The CSSS has been shown to have a high degree of internal consistency (Cronbach’s α = 0.78–0.89) and good construct validity among Japanese elementary school children [20]. In this study Cronbach’s α = 0.95.

### General mental health

Children’s general mental health was assessed using the self-, parent-, and teacher-rated Strengths and Difficulties Questionnaire (SDQ) [21]. The SDQ consists of four difficulty subscales (Emotional Symptoms, Conduct Problems, Hyperactivity/Inattention, and Peer Problems) and one strength subscale (Prosocial Behavior). Each subscale comprises 5 items with each item being scored on a 3-point scale. Subscale scores range from 0 to 10, with higher scores reflecting greater difficulty, while a higher prosocial subscale score indicates greater positive behavior. A total difficulties score (TDS) can be calculated by adding the four difficulty subscale scores together (range 0–40). The Japanese version of the questionnaire was used in this study [22].

### Autistic traits

Autistic traits were assessed with the Social Responsiveness Scale (SRS; [23]), a 65-item quantitative measure of autistic traits. Each item is scored on a 4-point scale with the total raw score ranging from 0 to 195, with higher scores reflecting greater autistic traits. This parent-rating scale has a continuous distribution in the general child population [24]. To examine whether the outcomes of this study differed by the degree of autistic traits, we created three groups using SRS T-scores; (i) children within the normal range (the ASD-Unlikely group, T-score ≤ 59), (ii) children with an increased number of autistic traits almost corresponding to subthreshold ASD (the ASD-Possible group, T-score ≥ 60 and ≤ 75), and (iii) children with the greatest autistic traits, corresponding to the threshold level (the ASD-Probable group, T-score ≥ 76). T-scores were calculated according to a Japanese norm stratified by gender [25].

GSESC-R, SDQ, and CSSS scores were obtained at T1, T2, and T3. SRS scores were obtained only at T1.

### Data analysis

Data from the three time points (T1, T2, and T3) were analyzed to assess changes from baseline using a linear mixed-effects model. The participants were included in the model as a random effect, while time, the evaluator (child, parent, or teacher), and the time-evaluator interaction were modeled as fixed effects, where the evaluator and interaction were included only for the SDQ. The three ASD groups divided by their SRS T-scores were also included in the above model. In these analyses, we estimated adjusted means for each level of fixed effects and conducted *t*-tests for the differences in adjusted means for T1–T2 and T1–T3.

A second analysis used a linear mixed-effects model to determine which factors contributed to the changes in the GSESC-R score from T1 to T2, and T3. The parent-rated SDQ (T1), teacher-rated SDQ (T1), and CSSS (T1) were omitted from the full analysis since initial analyses showed that they correlated poorly with change in the primary outcome. We included participants as a random effect and time (T2, T3) as a fixed effect. Candidates for fixed effects were gender, grade, autistic traits, the GSESC-R score (T1), and the 5 child-rated SDQ subscales (T1). Variable selection was conducted with a stepwise method using a *p*-value < 0.1 as a selection criterion.

All statistical analyses were performed using the SAS software program, version 9.4 (SAS Institute, Cary, NC, U.S.A) and SPSS Version 23.0 (IBM, Armonk, New York, U.S.A).

## Results

### Children’s characteristics at T1

Table 1 shows grade and gender information of the participants who were aged 9–12 years old. Information on the primary and secondary outcomes at T1 is presented in Table 2. Complete SRS data were collected for 285 children (51% male). The total raw SRS scores ranged from 2 to 97 (M = 34.1, SD = 18.0). The ASD-Unlikely group contained 84.6% of the children with complete SRS data, while the corresponding figures for the ASD-Possible and ASD-Probable groups were 11.9% and 3.5%, respectively.

**Table 1:** Characteristics of the study participants

	Participants (N = 396)		
Grade	Number (%)	Male	Female
4	280 (71)	144	136
5	73 (18)	41	32
6	43 (11)	22	21

**Table 2:** Primary and secondary outcome data at Baseline (T1) by autistic traits status

	Total (N = 396)			ASD-Unlikely (N = 241)			ASD-Possible (N = 34)			ASD-Probable (N = 10)		
	N	Mean (SD)	Range	N	Mean (SD)	Range	N	Mean (SD)	Range	N	Mean (SD)	Range
Primary Outcome												
Self-efficacy^a^	365^d^	26.08 (4.72)	12–36	222	26.57 (4.18)	15–36	26	23.12 (5.15)	12–30	10	22.30 (5.08)	15–32
Secondary Outcome												
Social skills^b^	185^e^	93.52 (16.92)	47–125	112	96.89 (15.90)	57–125	12	92.55 (15.10)	68–112	4	76.75 (12.42)	60–88
Mental health^c^												
Self-rated	364^f^	11.90 (5.96)	1–32	221	11.07 (5.21)	1–30	26	14.81 (6.43)	4–28	10	19.50 (6.72)	8–32
Parent-rated	311^ g^	7.90 (5.22)	0–28	240	6.54 (4.16)	0–22	34	13.06 (4.71)	4–24	9	19.67 (4.92)	11–28
Teacher-rated	194^ h^	5.80 (6.16)	0–26	118	4.47 (5.00)	0–24	12	5.83 (5.81)	1–17	4	13.00 (5.60)	6–18

*ASD* Autism spectrum disorder, *SD* standard deviation

^a^Assessed with the *GSESC-R* General Self-Efficacy Scale for Children-Revised

^b^Assessed with the *CSSS* Children’s Social Skills Scale

^c^Assessed with the *SDQ* Strengths and Difficulties Questionnaire

^d^There were 371 self-rated questionnaires for which informed consent was obtained, from which six with incomplete data were removed

^e^There were 194 teacher-rated questionnaires for which informed consent was obtained, from which nine with incomplete data were removed

^f^There were 371 self-rated questionnaires for which informed consent was obtained, from which seven with incomplete data were removed

^g^There were 317 parent-rated questionnaires for which informed consent was obtained, from which six with incomplete data were removed

^h^There were 194 teacher-rated questionnaires for which informed consent was obtained

### Changes in outcome measures for all children and the 3 ASD groups between T1–T3

#### Primary outcome: self-efficacy

We observed a significant difference in the adjusted mean change in the GSESC-R between T1 and T2 for all children (adjusted difference 0.49, 95% CI 0.03–0.94; p < 0.05) and between T1 and T3 (0.78, 95% CI 0.32–1.23; p < 0.001) (Table 3).

**Table 3:** Least square means and comparison with baseline data (T1) using a linear mixed-effects model

	T1		T2		p value^d^	T3		p value^e^
	Estimate	95% CI	Estimate	95% CI		Estimate	95% CI	
Primary outcome								
Self-efficacy^a^	26.05	(25.57–26.54)	26.54	(26.00–27.08)	0.04*	26.83	(26.29–27.37)	< 0.01**
Secondary Outcome								
Social skills^b^	93.45	(91.02–95.87)	96.56	(94.24–98.87)	< 0.01**	97.06	(94.69–99.42)	< 0.01**
Mental health^c^								
Self-rated	11.95	(11.34–12.56)	11.21	(10.57–11.86)	< 0.01**	10.20	(9.56–10.83)	< 0.01**
Parent-rated	8.13	(7.56–8.69)	8.16	(7.57–8.74)	0.88	7.71	(7.16–8.26)	0.04*
Teacher-rated	5.87	(5.07–6.68)	6.00	(5.15–6.85)	0.63	5.87	(5.03–6.70)	0.98

*CI* confidence interval

*T1* Baseline, *T2* immediately after the program finished, *T3* three months after the program finished

^*^p < .05, **p < .01

^a^Assessed with the *GSESC-R* General Self-Efficacy Scale for Children-Revised

^b^Assessed with the *CSSS* Children’s Social Skills Scale

^c^Assessed with the *SDQ* Strengths and Difficulties Questionnaire

^d^Comparing the T2 and baseline (T1) estimated scores

^e^Comparing the T3 and baseline (T1) estimated scores

**Fig. 1:**
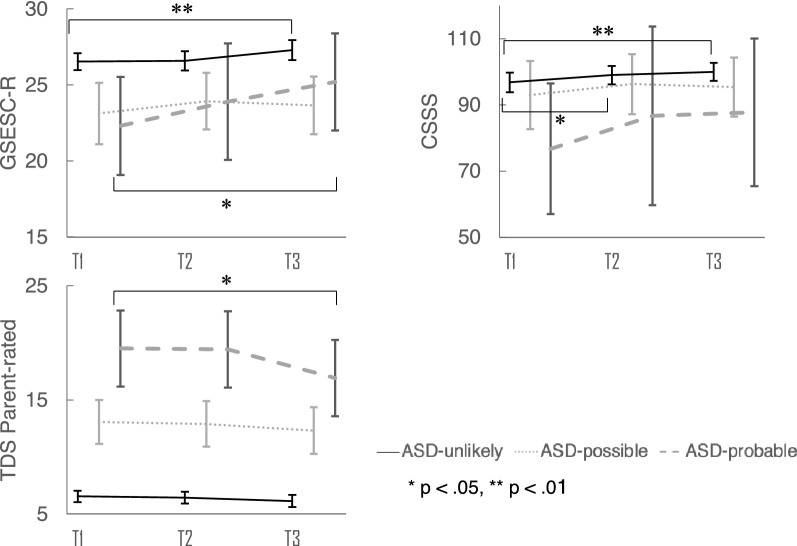
Changes in the outcome measures of the 3 ASD groups

The estimated adjusted means of the GSESC-R using a linear mixed-effects model and the results of *t*-tests for the differences in the adjusted means (T1-T2, T1-T3) among the 3 ASD groups are shown in Table 4 and Fig. 1. For the ASD-Unlikely group, there was a significant difference in the adjusted mean change in the GSESC-R score between T1 and T3 (0.77, 95% CI 0.19–1.23; p < 0.001). Similarly, for the ASD-Probable group, there was a significant difference in the GSESC-R score between T1 and T3 (2.90, 95% CI 0.03–5.77; p < 0.05). By contrast, for the ASD-Possible group, there were no significant across-time changes.

**Table 4:** An across-time comparison of the 3 ASD groups using a linear mixed-effects model

	ASD traits	T1		T2		p value^d^	T3		p value^e^
		Estimate	95%CI	Estimate	95%CI		Estimate	95%CI	
Primary outcome									
Self-efficacy^a^									
	ASD-Unlikely	26.53	(25.98–27.08)	26.59	(25.96–27.21)	0.84	27.29	(26.64–27.95)	< 0.01**
	ASD-Possible	23.12	(21.04–25.19)	23.93	(22.02–25.84)	0.26	23.65	(21.71–25.60)	0.38
	ASD-Probable	22.30	(18.67–25.93)	23.90	(19.55–28.25)	0.14	25.20	(21.59–28.81)	0.04*
Secondary outcome									
Social skills^b^									
	ASD-Unlikely	96.83	(93.84–99.83)	99.02	(96.26–101.8)	0.02*	100.00	(97.28–102.7)	< 0.01**
	ASD-Possible	93.03	(82.74–103.3)	96.31	(87.21–105.4)	0.07	95.38	(86.45–104.3)	0.38
	ASD-Probable	76.75	(56.99–96.51)	86.75	(59.73–113.8)	0.15	87.75	(65.42–110.1)	0.34
Mental health^c^									
Self-rated	ASD-Unlikely	11.11	(10.34–11.89)	10.55	(9.78–11.33)	0.07	9.08	(8.31–9.85)	< 0.01**
	ASD-Possible	14.69	(12.44–16.94)	13.28	(10.99–15.57)	0.14	13.04	(10.79–15.29)	0.07
	ASD-Probable	19.50	(14.70–24.30)	15.70	(10.90–20.50)	0.02*	14.90	(10.10–19.70)	< 0.01**
Parent-rated	ASD-Unlikely	6.54	(6.05–7.03)	6.44	(5.92–6.96)	0.66	6.14	(5.61- 6.67)	0.07
	ASD-Possible	13.06	(11.14–14.98)	12.89	(10.89–14.90)	0.84	12.31	(10.27–14.34)	0.37
	ASD-Probable	19.50	(16.16–22.84)	19.43	(16.09–22.77)	0.9*5*	16.90	(13.56–20.24)	0.03*
Teacher-rated	ASD-Unlikely	4.49	(3.62–5.35)	4.92	(4.05–5.78)	0.22	4.62	(3.76–5.49)	0.70
	ASD-Possible	5.26	(2.40–8.13)	5.96	(3.15–8.77)	0.54	6.73	(3.92–9.54)	0.21
	ASD-Probable	14.36	(7.46–21.26)	8.17	(1.24–15.10)	0.02*	10.36	(3.42–17.29)	0.08

*ASD* Autism Spectrum Disorder, *CI* confidence interval

*T1*: Baseline, *T2*: immediately after the program, *T3*: three months after the program finished

^*^p < .05, **p < .01

^a^Assessed with the *GSESC-R* General Self-Efficacy Scale for Children-Revised

^b^Assessed with the *CSSS* Children’s Social Skills Scale

^c^Assessed with the *SDQ* Strengths and Difficulties Questionnaire

^d^Comparing the T2 and baseline (T1) estimated scores

^e^Comparing the T3 and baseline (T1) estimated scores

#### Secondary outcome: social skills and problem behaviors (CSSS)

There was a significant increase in the CSSS adjusted mean score for all children between T1 and T2 (3.11, 95% CI 1.69–4.53; p < 0.001) and between T1 and T3 (3.61, 95% CI 2.14–5.08; p < 0.001) (Table 3).

Among the ASD groups (Table 4; Figure 1), there was a significant change in CSSS adjusted means only for the ASD-Unlikely group between T1 and T2 (2.18, 95% CI 0.38–3.99; p < 0.05) and between T1 and T3 (3.17, 95% CI 1.40–4.94; p < 0.001).

### Secondary outcome: general mental health (SDQ)

For the whole group there were significant differences in the adjusted mean change in the self-rated SDQ TDS between T1 and T2 (− 0.74, 95% CI − 1.27 to − 0.20; p < 0.001), between T1 and T3 (− 1.75, 95% CI − 2.22 to − 1.28; p < 0.001), and in the parent-rated SDQ TDS between T1 and T3 (− 0.42, 95% CI − 0.82 to − 0.01; p < 0.05) (Table 3). However, there were no significant changes in the teacher-rated SDQ TDS scores.

There were significant improvements in each of the 4 SDQ self-rated difficulty subscale scores, whereas only the Conduct Problems subscale score improved for parent ratings. In contrast, there were no significant changes for any of the teacher-rated difficulty subscale scores. For the Prosocial Behavior subscale, there were significant improvements in child ratings between T1 and T2, and teacher ratings between T1 and T2, and T1 and T3 (see Additional file 3).

By ASD group, outcome changes were observed in both the ASD-Unlikely and the ASD-Probable groups. For the ASD-Unlikely group, the adjusted mean of the self-rated SDQ TDS improved between T1 and T3 (− 2.04, 95% CI − 2.65 to − 1.42; p < 0.001) (Table 4). For the ASD-Probable group, the adjusted mean of the self-rated SDQ TDS improved between T1 and T2 (− 3.80, 95% CI − 6.94 to − 0.66; p < 0.05), and T1 and T3 (− 4.60, 95% CI − 7.74 to − 1.46; p < 0.001). In addition, the adjusted mean of the parent-rated SDQ TDS for the ASD-Probable group improved between T1 and T3 (− 2.60, 95% CI − 4.95 to − 0.25; p < 0.05), while those of the teacher-rated SDQ TDS improved between T1 and T2 (− 6.19, 95% CI − 10.7 to − 1.69; p < 0.05) (Table 4; Fig. 1). By contrast, for the ASD-Possible group, there were no significant changes in the self-rated, parent-rated, or teacher-rated SDQ TDS. The results for the SDQ subscales for each ASD group by different raters are shown in Additional file 4.

### Variables associated with the changes in the primary outcome

Table 5 presents the regression coefficients for the variables associated with changes in the GSESC-R scores at T2 and T3 compared with T1. Grade, T1 GSESC-R, and the T1 child-rated Hyperactivity/Inattention SDQ subscale score predicted changes in the GSESC-R (T1-T2-T3). Variables such as gender and the SRS score were not associated with across-time changes in the primary outcome.

**Table 5:** Regression coefficients associated with change in the GSESC-R score at T2 and T3 compared to T1

	ΔGSESC-R			
	Estimate	(95% CI)	t value	p value
Intercept	17.95	(13.98 to 21.93)	8.88	< 0.01
Time (T2)	− 0.41	(− 0.83 to 0.00)	− 1.96	0.05
Grade	− 1.06	(− 1.61 to − 0.52)	− 3.83	< 0.01
GSESC-R at T1	− 0.43	(− 0.52 to − 0.33)	− 8.97	< 0.01
Hyperactivity at T1	− 0.34	(− 0.53 to − 0.16)	− 3.60	< 0.01

*GSESC-R* General Self-Efficacy Scale for Children-Revised

## Discussion

### Change in the primary outcome

This study examined whether a school-based universal intervention program, the Up2-D2, would increase children’s self-efficacy as a primary outcome and improve social skills and mental health as secondary outcomes. Results showed that children who received the Up2-D2 program at school not only experienced a reduction in their general mental health-related symptoms but also an improvement in self-efficacy and social skills immediately after the program, which persisted three months later.

Our positive results may be related to the adaptation of the intervention to the school environment including the involvement of teachers in the delivery of the program, which has been pointed out as a key feature in achieving positive results in universal intervention programs [10]. In the present study, in order to facilitate the seamless implementation of the program, we translated the program into ‘teaching plans’, created visual materials for teachers to use when implementing the program in their classrooms, and set the program's delivery time to coincide with the school's instructional time.

The use of cartoon characters and a workbook as a main feature of the Up2-D2 might have provided an accessible storyline for the children, which may have made it more enjoyable for them too. An earlier study that implemented a CBT-based universal program, using cartoon characters, reported that resilience improved in 3rd to 4th grade children after the completion of the program [26]. There are similarities and differences between that study [26] and ours; both used a CBT-based universal approach and had a positive impact on resilience. On the other hand, Yamamoto et al.’s study [26] had a control group, their participants were younger, the researchers rather than teachers implemented the program, and the program was conducted in only one city. This may suggest that the Up2-D2 program might be beneficial for even younger children, although these studies cannot be directly compared.

### Changes in the secondary outcomes

In our study, robust improvements were observed in teacher-rated social skills and self-rated general mental health, which is consistent with the results of existing research. In terms of social skills, a meta-analysis of school-based universal-level programs for children aged 5–18 by Durlak et al. [27] found a significant effect size for ‘positive social behavior’. A systematic review and meta-analysis of school-based universal-level programs focusing on ‘resilience-enhancing’ found a significant improvement in depression, anxiety, and general psychological stress for CBT programs but not for non-CBT programs [9] for children aged 5–18. Furthermore, that meta-analysis also provided evidence of an age effect; effects on anxiety and general psychological stress were found only for children (aged 5–10 years old) but not for adolescents (aged 11–18 years old) [9]. In our study, children younger than 10 years old accounted for 71% of the total sample. Thus, the positive outcome in general mental health might be explained by the fact that a majority of our sample were elementary school children.

### Influence of autistic traits

One of the reasons why previous studies may have found universal programs to be less effective is because of what has been termed ‘the ceiling effect’ [6, 28], where the majority of individuals in mainstream classes are low-risk children. Among our participants, the prevalence of autistic traits was similar to that found in a previous Japanese representative sample. Specifically, 3.5% of the whole sample in our study had a threshold level of autistic traits, which corresponds closely to the figure—3.6%—identified in a national survey of Japanese schoolchildren in mainstream classes conducted by the Ministry of Education, Culture, Sports, Science and Technology [29]. Children with subthreshold-level autistic traits comprised 11.9% of the whole sample, which is also very similar to the figure of 10.9% reported in an earlier Japanese study [24]. Furthermore, children with higher autistic traits had lower self-efficacy and more mental health problems at baseline than children with few or no traits in our study. Such an association between autistic traits and mental health problems is also consistent with earlier findings from a Japanese community-based study [17]. Thus, while there are a certain percentage of children at high-risk for ASD in school settings, there are also children with a lower risk in the same classes. This suggests that in order for a school-based intervention program to be educationally inclusive and truly beneficial, it needs to be effective for both types of children.

Given this, the current study examined whether changes in outcomes differed across groups with different levels of autistic traits using a linear mixed-effects model. Children with the highest levels of autistic traits showed improvements in self-efficacy and general mental health problems, as did children with few or no autistic traits. These results indicate that the Up2-D2 program has the potential to promote positive attitudes and improved mental health in elementary school children, regardless of their level of autistic traits. To the best of our knowledge, no prior studies have examined the impact of universal programs on children with autistic traits, although the effects of selective programs on children with a diagnosis of autism have been extensively reported [30, 31]. Given the seemingly high prevalence of ASD at threshold/subthreshold levels, there is likely to be a lack of manpower within schools to implement selective programs for all those children with higher autistic traits [12]. In connection with this, our findings raise the possibility that teacher-administered, classroom-wide mental health programs might be affordable and helpful for children with and without autistic traits.

Autistic traits as measured by the SRS score were not associated with the primary outcome, changes in the level of self-efficacy, during the study period. Rather, younger age, lower self-efficacy, and fewer self-rated inattention/hyperactivity symptoms at baseline were associated with a greater change in self-efficacy. The finding that younger children may benefit more suggests that it may be preferable to start implementation of the program during middle childhood, although further research is needed on the feasibility and acceptability of the intervention at that age. It is also understandable that children with hyperactivity and inattention symptoms may be less likely to benefit from the program. Children who are hyperactive and inattentive are known to have poor academic and educational outcomes even if they do not have a diagnosis of attention-deficit/hyperactivity disorder (ADHD) [32], and need individual support in the classroom [33]. Indeed, children with ADHD often have comorbid ASD symptoms [34, 35]. In this study, the self-rated inattention and hyperactivity scores of children with subthreshold-level autistic traits at T1 were ranged beyond the upper end of those of the unlikely group. It can be speculated that one of the reasons why we found fewer changes in the primary outcome of the group with subthreshold-level autistic traits is that higher levels of hyperactivity/inattention in this group could have affected the results. Rigorous research is needed in the future to identify factors related to the intervention outcome and to enable effective support to be provided for these children.

## Limitations

This study has several limitations. Regarding the study design, the sample size was small given the diversity of the children, no control group was set up, and the follow-up period was short. In addition, we cannot discount the possibility that there might have been important differences between those families/parents (55%) that were willing to let their children participate in the study and those that did not (45%). Such potential bias should be kept in mind when interpreting the results. Regarding the SDQ, self-ratings were obtained from children younger than 11 years of age. This may have been problematic as even for children over 11 years of age, the reliability and validity of self-rated mental health measures among ASD populations have yet to be established, although high-functioning individuals with ASD may be able to report their own psychiatric symptoms to a certain degree [36]. However, the changes in outcome associated with self-rated scores were in parallel with those from parent-rated scores in our study, which suggests that our findings from self-rated scores may be reliable. Future RCTs with a larger sample size and a longer research and follow-up period are necessary to determine the efficacy of the universal program for high- and low-risk children in mainstream classes.

## Conclusions

The current study found that the implementation of a newly developed school-based universal program in elementary school mainstream classes was associated with positive changes across multiple domains, including self-efficacy, social skills, and mental health problems. Furthermore, the level of autistic traits did not affect the degree of change in self-efficacy after the program. This suggests that the universal implementation of the program may result in a wide range of positive benefits for children both at high- and low-risk for mental health problems. However, future rigorous research is needed to confirm this hypothesis from various perspectives, for such outcomes as a longer-term follow-up beyond three months, referral rates to child mental health services, and cost-effectiveness.

## Supplementary Information

**Additional file 1**: The data collection process. Among all children recruited, the number of consenting children, parents, and teachers is shown.

**Additional file 2**: Components of the Up2-D2. The table shows the contents of each session of the Up2-D2 program.

**Additional file 3**: Least square means and a comparison with the SDQ subscales at baseline using a linear mixed-effects model.

**Additional file 4**: An across-time comparison of the SDQ subscale scores for the 3 ASD groups using a linear mixed-effects model.

## Data Availability

The datasets generated and analyzed during the current study are not publicly available but are available from the corresponding author upon reasonable request.

## Abbreviations

RCT: Randomized controlled trial
SMD: Standardized Mean Difference
Up2-D2: Universal Unified Prevention Program for Diverse Disorders
CBT: Cognitive behavior therapy
ASD: Autism spectrum disorder
GSESC-R: General Self-Efficacy Scale for Children-Revised
CSSS: Children’s Social Skills Scale
SDQ: Strengths and Difficulties Questionnaire
TDS: Total difficulties score
SRS: Social Responsiveness Scale
ADHD: Attention-deficit/hyperactivity disorder
