# Supplementary material for: Changes in self-efficacy in Japanese school-age children with and without high autistic traits after the Universal Unified Prevention Program: a single-group pilot study
Source: Child Adolesc Psychiatry Ment Health. 2021 Aug 26;15:42. doi: 10.1186/s13034-021-00398-y (PMC8390243; doi:10.1186/s13034-021-00398-y)
Supplement: Supplementary file 1 — Additional file 1: The data collection process. Among all children recruited, the number of consenting children, parents, and teachers is shown. [file 13034_2021_398_MOESM1_ESM.docx]

**Additional file 1:** The process of data collection

Students who received the program†

n=715

Child-rated data

(n=365)

Teacher-rated data*

(n=194)

Parent-rated data

(n=311)

Informed consent for self-rated questionnaire data (n=371)

Informed consent for teacher-rated questionnaire data (n=371)

Informed consent for parent-rated questionnaire data (n=317)

†All the children received the Up2-D2 program as a part of the regular curriculum.

* According to a predetermined rule, teachers rated 10 children (5 boys, 5 girls) who were randomly chosen by the research team from among those in each class for whom parents gave written consent. In 8 classes where fewer than 10 parents gave consent teachers provided information for all of the children whose parents did give consent.
