# Supplementary material for: Changes in self-efficacy in Japanese school-age children with and without high autistic traits after the Universal Unified Prevention Program: a single-group pilot study
Source: Child Adolesc Psychiatry Ment Health. 2021 Aug 26;15:42. doi: 10.1186/s13034-021-00398-y (PMC8390243; doi:10.1186/s13034-021-00398-y)
Supplement: Supplementary file 2 — Additional file 2: Components of the Up2-D2. The table shows the contents of each session of the Up2-D2 program. [file 13034_2021_398_MOESM2_ESM.docx]

Additional file 2 Components of the Up2-D2

| Session No. | Aim | Component |
| --- | --- | --- |
| 1 | Introduction to the program | Psychoeducation |
| 2 | Exploring pleasant events | Behavioral activation |
| 3 | Learning about kind words | Social skills training |
| 4 | Learning about assertive skills | Social skills training |
| 5 | Relaxation training | Relaxation |
| 6 | Identifying one’s own and others’ strengths | Strength work |
| 7 | Discovery of one’s own cognition | Cognitive restructuring |
| 8 | Challenging unhelpful thoughts | Cognitive restructuring |
| 9 | Preparing behavioral challenges | Exposure |
| 10 | Building-up behavioral challenges | Exposure |
| 11 | Learning about problem-solving skills | Problem solving |
| 12 | Conclusion | Review and conclusion |
