# Supplementary material for: Changes in self-efficacy in Japanese school-age children with and without high autistic traits after the Universal Unified Prevention Program: a single-group pilot study
Source: Child Adolesc Psychiatry Ment Health. 2021 Aug 26;15:42. doi: 10.1186/s13034-021-00398-y (PMC8390243; doi:10.1186/s13034-021-00398-y)
Supplement: Supplementary file 3 — Additional file 3: Least square means and a comparison with the SDQ subscales at baseline using a linear mixed-effects model. [file 13034_2021_398_MOESM3_ESM.docx]

Additional file 3 Least square means and a comparison with the SDQ subscales at baseline using a linear mixed-effects model

|  | T1 | | T2 | |  | T3 | |  |
| --- | --- | --- | --- | --- | --- | --- | --- | --- |
| **SDQ Subscales** | Estimate | 95%CI | Estimate | 95%CI | P value^a^ | Estimate | 95%CI | P value^b^ |
| Emotional Symptoms |  |  |  |  |  |  |  |  |
| Self-rated | 3.34 | (3.09 - 3.59) | 3.10 | (2.84 - 3.37) | 0.04* | 2.87 | (2.60 - 3.13) | < 0.01** |
| Parent-rated | 1.59 | (1.40 - 1.78) | 1.51 | (1.32 - 1.70) | 0.36 | 1.54 | (1.35 - 1.73) | 0.54 |
| Teacher-rated | 1.06 | (0.82 - 1.29) | 1.15 | (0.91 - 1.39) | 0.41 | 1.06 | (0.82 - 1.30) | 0.95 |
| Conduct Problems |  |  |  |  |  |  |  |  |
| Self-rated | 2.25 | (2.08 - 2.43) | 2.19 | (2.01 - 2.37) | 0.51 | 1.93 | (1.77 - 2.10) | < 0.01** |
| Parent-rated | 1.99 | (1.81 - 2.18) | 1.94 | (1.76 - 2.12) | 0.50 | 1.80 | (1.63 - 1.97) | 0.02* |
| Teacher-rated | 0.90 | (0.70 - 1.09) | 0.96 | (0.76 - 1.17) | 0.37 | 0.97 | (0.77 - 1.17) | 0.34 |
| Hyperactivity/Inattention |  |  |  |  |  |  |  |  |
| Self-rated | 3.79 | (3.55 - 4.03) | 3.57 | (3.31 - 3.83) | 0.04* | 3.27 | (3.03 - 3.52) | < 0.01** |
| Parent-rated | 3.03 | (2.79 - 3.27) | 3.13 | (2.88 - 3.37) | 0.28 | 2.89 | (2.65 - 3.13) | 0.13 |
| Teacher-rated | 2.61 | (2.23 - 2.98) | 2.70 | (2.30 - 3.09) | 0.44 | 2.59 | (2.21 - 2.98) | 0.91 |
| Peer problems |  |  |  |  |  |  |  |  |
| Self-rated | 2.59 | (2.40 - 2.77) | 2.35 | (2.16 - 2.53) | 0.01* | 2.15 | (1.97 - 2.33) | < 0.01** |
| Parent-rated | 1.52 | (1.35 - 1.68) | 1.57 | (1.39 - 1.75) | 0.51 | 1.52 | (1.35 - 1.69) | 0.97 |
| Teacher-rated | 1.31 | (1.07 - 1.55) | 1.28 | (1.05 - 1.51) | 0.72 | 1.31 | (1.07 - 1.55) | 0.99 |
| Prosocial Behaviour |  |  |  |  |  |  |  |  |
| Self-rated | 6.52 | (6.32 - 6.72) | 6.74 | (6.52 - 6.95) | 0.02* | 6.62 | (6.42 - 6.83) | 0.27 |
| Parent-rated | 6.73 | (6.49 - 6.96) | 6.44 | (6.21 - 6.67) | < 0.01** | 6.44 | (6.20 - 6.68) | 0.01* |
| Teacher-rated | 6.96 | (6.62 - 7.30) | 7.23 | (6.88 - 7.57) | 0.03* | 7.29 | (6.96 - 7.63) | 0.01* |

SDQ: Strengths and Difficulties Questionnaire; CI: Confidence interval

*p < .05, **p < .01

^a^Comparing the T2 and baseline (T1) estimated scores

^b^Comparing the T3 and baseline (T1) estimated scores

T1: Baseline, T2: Immediately after the program finished, T3: Three months after the program finished.
