# Supplementary material for: Changes in self-efficacy in Japanese school-age children with and without high autistic traits after the Universal Unified Prevention Program: a single-group pilot study
Source: Child Adolesc Psychiatry Ment Health. 2021 Aug 26;15:42. doi: 10.1186/s13034-021-00398-y (PMC8390243; doi:10.1186/s13034-021-00398-y)
Supplement: Supplementary file 4 — Additional file 4: An across-time comparison of the SDQ subscale scores for the 3 ASD groups using a linear mixed-effects model. [file 13034_2021_398_MOESM4_ESM.docx]

Additional file 4 An across-time comparison of the SDQ subscale scores for the 3 ASD groups using a linear mixed-effects model

|  |  | T1 | | T2 | |  | T3 | |  |
| --- | --- | --- | --- | --- | --- | --- | --- | --- | --- |
|  | ASD-traits | Estimate | 95%CI | Estimate | 95%CI | P value^a^ | Estimate | 95%CI | P value^b^ |
| **SDQ subscales** |  |  |  |  |  |  |  |  |  |
| Emotional Symptoms |  |  |  |  |  |  |  |  |  |
| Self-rated | ASD-Unlikely | 3.20 | (2.89 - 3.51) | 3.01 | (2.70 - 3.32) | 0.20 | 2.59 | (2.28 - 2.90) | < 0.01** |
|  | ASD-Possible | 3.49 | (2.60 - 4.38) | 3.93 | (3.03 - 4.83) | 0.32 | 3.73 | (2.84 - 4.62) | 0.59 |
|  | ASD-Probable | 6.60 | (4.83 - 8.37) | 4.00 | (2.23- 5.77) | < 0.01** | 4.20 | (2.43 - 5.97) | < 0.01** |
| Parent-rated | ASD-Unlikely | 1.25 | (1.08 - 1.43) | 1.18 | (0.99 - 1.37) | 0.46 | 1.21 | (1.01 - 1.40) | 0.63 |
|  | ASD-Possible | 2.65 | (1.99 - 3.31) | 2.80 | (2.10 - 3.50) | 0.66 | 2.94 | (2.23 - 3.66) | 0.40 |
|  | ASD-Probable | 4.65 | (3.10 - 6.20) | 3.55 | (1.99 - 1.37) | 0.14 | 3.12 | (1.01 - 1.40) | 0.04* |
| Teacher-rated | ASD-Unlikely | 0.56 | (0.30 - 0.83) | 0.85 | (0.59 - 1.11) | 0.03* | 0.75 | (0.49 - 1.01) | 0.15 |
|  | ASD-Possible | 2.01 | (0.58 - 0.82) | 1.97 | (0.57 - 0.81) | 0.94 | 2.15 | (0.57 – 0.99) | 0.80 |
|  | ASD-Probable | 1.61 | (-0.65 - 3.87) | 0.75 | (-1.52 - 3.02) | 0.37 | 0.43 | (-1.84 - 2.70) | 0.23 |
| Conduct Problems |  |  |  |  |  |  |  |  |  |
| Self-rated | ASD-Unlikely | 2.09 | (1.87 - 2.31) | 2.05 | (1.83 - 2.27) | 0.67 | 1.74 | (1.52 - 1.96) | < 0.01** |
|  | ASD-Possible | 2.52 | (1.92 - 3.11) | 1.81 | (1.20 - 2.42) | 0.02* | 2.01 | (1.42 - 2.61) | 0.10 |
|  | ASD-Probable | 3.70 | (2.70 - 4.70) | 3.00 | (2.00 - 4.00) | 0.01* | 2.80 | (1.80 - 3.80) | < 0.01** |
| Parent-rated | ASD-Unlikely | 1.59 | (1.43 - 1.76) | 1.55 | (1.37 - 1.73) | 0.61 | 1.38 | (1.20 - 1.57) | 0.02* |
|  | ASD-Possible | 2.88 | (2.36 - 3.41) | 2.42 | (1.86 - 2.98) | 0.10 | 2.56 | (1.98 - 3.13) | 0.26 |
|  | ASD-Probable | 4.23 | (2.58 - 5.89) | 3.97 | (2.30 - 5.63) | 0.57 | 3.45 | (1.79 - 5.12) | 0.11 |
| Teacher-rated | ASD-Unlikely | 0.75 | (0.53 - 0.97) | 0.75 | (0.53 - 0.97) | 0.98 | 0.82 | (0.60 - 1.04) | 0.51 |
|  | ASD-Possible | 0.25 | (-0.14 - 0.64) | 0.27 | (-0.11 - 0.65) | 0.92 | 0.42 | (0.05 - 0.80) | 0.37 |
|  | ASD-Probable | 1.41 | (-1.60 - 4.41) | 1.53 | (-1.47 - 4.53) | 0.86 | 1.16 | (-1.84 - 4.16) | 0.72 |
| Hyperactivity/Inattention |  |  |  |  |  |  |  |  |  |
| Self-rated | ASD-Unlikely | 3.43 | (3.11 - 3.74) | 3.32 | (3.01 - 3.64) | 0.38 | 2.84 | (2.52 - 3.15) | < 0.01** |
|  | ASD-Possible | 5.03 | (4.17 - 5.90) | 4.56 | (3.70 - 5.43) | 0.15 | 4.32 | (3.45 - 5.18) | 0.03* |
|  | ASD-Probable | 5.40 | (3.50 - 7.30) | 5.80 | (3.90 - 7.70) | 0.59 | 5.20 | (3.30 - 7.10) | 0.79 |
| Parent-rated | ASD-Unlikely | 2.53 | (2.29 - 2.77) | 2.50 | (2.25 - 2.75) | 0.77 | 2.34 | (2.09 - 2.60) | 0.06 |
|  | ASD-Possible | 4.50 | (3.63 - 5.37) | 4.73 | (3.83 - 5.64) | 0.51 | 4.13 | (3.21 - 5.05) | 0.30 |
|  | ASD-Probable | 6.13 | (4.67 - 7.59) | 6.67 | (5.21 - 8.14) | 0.37 | 5.89 | (4.42 - 7.35) | 0.68 |
| Teacher-rated | ASD-Unlikely | 2.12 | (1.71 - 2.52) | 2.27 | (1.87 - 2.68) | 0.30 | 2.05 | (1.64 - 2.45) | 0.66 |
|  | ASD-Possible | 1.49 | (0.03 - 2.95) | 1.82 | (0.39 - 3.26) | 0.54 | 1.87 | (0.43 - 3.30) | 0.49 |
|  | ASD-Probable | 4.92 | (2.96 - 6.87) | 2.59 | (0.63 - 4.55) | < 0.01** | 4.08 | (2.12 - 6.04) | 0.27 |
| Peer problems |  |  |  |  |  |  |  |  |  |
| Self-rated | ASD-Unlikely | 2.41 | (2.19 - 2.63) | 2.20 | (1.98 - 2.41) | 0.04* | 1.95 | (1.74 - 2.17) | < 0.01** |
|  | ASD-Possible | 3.71 | (2.88 - 4.54) | 2.97 | (2.14 - 3.80) | 0.02* | 3.00 | (2.17 - 3.83) | 0.03* |
|  | ASD-Probable | 3.80 | (1.97 - 5.63) | 2.90 | (1.07 - 4.73) | 0.22 | 2.70 | (0.87 - 4.53) | 0.13 |
| Parent-rated | ASD-Unlikely | 1.15 | (1.00 - 1.30) | 1.21 | (1.05 - 1.38) | 0.45 | 1.22 | (1.05 - 1.39) | 0.38 |
|  | ASD-Possible | 3.03 | (2.30 - 3.76) | 2.97 | (2.21 - 3.72) | 0.84 | 2.63 | (1.86 - 3.39) | 0.17 |
|  | ASD-Probable | 4.60 | (3.41 - 5.78) | 4.93 | (3.75 - 6.12) | 0.50 | 4.49 | (3.31 - 5.67) | 0.82 |
| Teacher-rated | ASD-Unlikely | 1.04 | (0.79 - 1.29) | 1.06 | (0.81 - 1.31) | 0.86 | 1.02 | (0.77 - 1.27) | 0.87 |
|  | ASD-Possible | 1.47 | (0.33 - 2.62) | 1.71 | (0.58 - 2.85) | 0.58 | 2.11 | (0.97 - 3.24) | 0.15 |
|  | ASD-Probable | 4.41 | (1.80 - 7.01) | 3.18 | (0.56 - 5.81) | 0.23 | 3.38 | (0.75 - 6.00) | 0.31 |
| Prosocial Behaviour |  |  |  |  |  |  |  |  |  |
| Self-rated | ASD-Unlikely | 6.60 | (6.35 - 6.85) | 6.61 | (6.36 - 6.87) | 0.92 | 6.71 | (6.45 - 6.96) | 0.40 |
|  | ASD-Possible | 5.42 | (4.69 - 6.14) | 5.78 | (4.94 - 6.62) | 0.22 | 5.66 | (4.76 - 6.56) | 0.42 |
|  | ASD-Probable | 6.40 | (5.28 - 7.52) | 6.50 | (5.31 - 7.69) | 0.70 | 6.90 | (5.34 - 8.46) | 0.40 |
| Parent-rated | ASD-Unlikely | 6.98 | (6.72 - 7.23) | 6.67 | (6.39 - 6.95) | 0.02* | 6.67 | (6.38 - 6.96) | 0.02* |
|  | ASD-Possible | 5.82 | (4.96 - 6.67) | 5.93 | (5.32 - 6.55) | 0.75 | 5.70 | (4.84 - 6.57) | 0.78 |
|  | ASD-Probable | 5.77 | (4.31 - 7.23) | 5.28 | (3.77 - 6.79) | 0.25 | 4.94 | (3.33 - 6.56) | 0.05 |
| Teacher-rated | ASD-Unlikely | 7.05 | (6.61 - 7.50) | 7.34 | (6.90 - 7.78) | 0.09 | 7.44 | (7.01 - 7.86) | 0.02* |
|  | ASD-Possible | 6.97 | (5.84 - 8.10) | 7.28 | (5.71 - 8.84) | 0.49 | 7.24 | (6.04 - 8.43) | 0.61 |
|  | ASD-Probable | 4.17 | (3.02 - 5.31) | 6.44 | (5.21 - 7.68) | < 0.01** | 5.95 | (4.58 - 7.33) | < 0.01** |

ASD: Autism Spectrum Disorder; SDQ: Strengths and Difficulties Questionnaire; CI: confidence interval

*p < .05, **p < .01

^a^Comparing the T2 and baseline (T1) estimated scores

^b^Comparing the T3 and baseline (T1) estimated scores

T1: Baseline, T2: Immediately after the program finished, T3: Three months after the program finished.
